# Neuro-Ophthalmological Findings in Friedreich’s Ataxia

**DOI:** 10.3390/jpm11080708

**Published:** 2021-07-23

**Authors:** Pilar Rojas, Rosa de Hoz, Manuel Cadena, Elena Salobrar-García, José A. Fernández-Albarral, Inés López-Cuenca, Lorena Elvira-Hurtado, José L. Urcelay-Segura, Juan J. Salazar, José M. Ramírez, Ana I. Ramírez

**Affiliations:** 1 Hospital General Universitario Gregorio Marañón, Instituto Oftálmico de Madrid, 28007 Madrid, Spain; pilar.rojas.lozano@gmail.com (P.R.); cadenamd@gmail.com (M.C.); joseluis.urcelay@salud.madrid.org (J.L.U.-S.); 2 Instituto de Investigaciones Oftalmológicas Ramón Castroviejo, Universidad Complutense de Madrid, IdISSC, 28040 Madrid, Spain; rdehoz@med.ucm.es (R.d.H.); elenasalobrar@med.ucm.es (E.S.-G.); joseaf08@ucm.es (J.A.F.-A.); inelopez@ucm.es (I.L.-C.); marelvir@ucm.es (L.E.-H.); jjsalazar@med.ucm.es (J.J.S.); 3 Departamento de Inmunología, Oftalmología y ORL, Facultad de Óptica y Optometría, Universidad Complutense de Madrid, 28037 Madrid, Spain; 4 Departamento de Inmunología, Oftalmología y ORL, Facultad de Medicina, Universidad Complutense de Madrid, 28040 Madrid, Spain

**Keywords:** Friedreich ataxia, FRDA, neurodegeneration, neurological disability, eye, retina

## Abstract

Friedreich ataxia (FRDA) is a progressive neurodegenerative disease caused by a severe autosomal recessive genetic disorder of the central nervous (CNS) and peripheral nervous system (PNS), affecting children and young adults. Its onset is before 25 years of age, with mean ages of onset and death between 11 and 38 years, respectively. The incidence is 1 in 30,000–50,000 persons. It is caused, in 97% of cases, by a homozygous guanine-adenine-adenine (GAA) trinucleotide mutation in the first intron of the frataxin (*FXN*) gene on chromosome 9 (9q13–q1.1). The mutation of this gene causes a deficiency of frataxin, which induces an altered inflow of iron into the mitochondria, increasing the nervous system’s vulnerability to oxidative stress. The main clinical signs include spinocerebellar ataxia with sensory loss and disappearance of deep tendon reflexes, cerebellar dysarthria, cardiomyopathy, and scoliosis. Diabetes, hearing loss, and pes cavus may also occur, and although most patients with FRDA do not present with symptomatic visual impairment, 73% present with clinical neuro-ophthalmological alterations such as optic atrophy and altered eye movement, among others. This review provides a brief overview of the main aspects of FRDA and then focuses on the ocular involvement of this pathology and the possible use of retinal biomarkers.

## 1. Introduction: Overview of Friedreich Ataxia Disease

Within the spinocerebellar ataxias, Friedreich’s ataxia (FRDA) is the most common autosomal recessive hereditary form [1,2]. In 97% of cases, it is caused by a homozygous guanine-adenine-adenine (GAA) trinucleotide mutation in the first intron of the frataxin gene (*FXN*) on chromosome 9. (9q13–q1.1) [2,3,4,5,6,7,8]. This mutation was first identified in 1996 [2]. Very few FRDA patients (approximately 2% to 4%) are compound heterozygotes with a GAA expansion on one allele and a deletion on the other [9], with the clinical phenotype being very similar to homozygous GAA; however, optic atrophy may be more common [9]. The *FXN* gene encodes frataxin (*FXN*), a small, long mitochondrial protein that, in the human body, is found in high concentrations in the cells of the heart, spinal cord, liver, pancreas, and voluntary movement muscles [10]. The mutation in this gene causes a deficiency of *FXN*, which leads to (i) insufficient biosynthesis of iron-sulfur (Fe-S) groups that are necessary for mitochondrial electron transport and the functional assembly of aconitase, and (ii) impaired iron metabolism in the cell [8]. The effect of the *FXN* mutation leads to an altered iron supply to the mitochondria, increasing the vulnerability of the nervous system to oxidative stress, including the visual pathway [11,12,13].

### 1.1. Epidemiology

The incidence of FRDA worldwide is 1 in 30,000 to 50,000 people, being more common in Caucasians [1,4,6,7,9,11,14]. Specifically in Western Europe, the incidence ranges from 1 in 20,000 to 125,000. The GAA trinucleotide repeat expansion only occurs in European, North African, Middle Eastern, and Indian individuals and is not found in far Eastern, sub-Saharan African, Native American, or Australian individuals [7].

The prevalence is approximately 1 in 50,000 individuals [1]. In 75% of cases, the onset of the disease is usually before 25 years of age, and there is no sex predilection [5,6].

### 1.2. Clinic

In FRDA, there is a specific susceptibility of organs and tissues to systemic *FXN* deficiency [8]. The pathology and clinical presentation of FRDA are unique, although it has quite a considerable overlap with hereditary vitamin E deficiency, which is included in its differential diagnosis [14,15].

The typical form of FRDA is clinically defined as having autosomal recessive inheritance with onset before 25 years of age, with mean ages of onset and death of approximately 11 and 38 years, respectively [16].

There is neurological involvement of both the peripheral nervous system (PNS) and the central nervous system (CNS) in this pathology. The typical clinical signs include progressive gait ataxia with proprioceptive sensory impairment, particularly loss of vibratory sense and proprioception, and limb atrophy associated with first and second motor neuron involvement [8]. In addition, it may present with combined symptoms such as loss of muscle tone, spasticity, depressed deep tendon reflexes, or extensor plantar responses (Babinski). It also presents with dysmetria, cerebellar dysarthria, dysphagia, weakness, complex oculomotor disturbances, visual loss, and hearing deficits [16]. Electrophysiological studies of FRDA demonstrate that motor potentials might be normal in an initial stage, but in late stages, patients present both sensitive and motor neuropathy. In addition, magnetic resonance imaging (MRI) of the brain is generally normal at disease onset [17]. In late stages with advanced disease, atrophy of the cervical spinal cord and cerebellum may be observed [18]. Atrophy of the superior cerebellar peduncle, the main outflow tract of the dentate nucleus, may also be seen [19]. Cervical spinal cord size correlates with disease severity as measured by the Friedreich Ataxia Rating Scale [20].

FRDA is an insidious disease, with a deterioration of the cerebellar, posterior columns, nerves, and a gradual decline in muscle function; most patients lose the capacity to ambulate 10 to 15 years from diagnosis, and thereafter, requiring a wheelchair [8]. The disease does not progress either constantly or linearly and may vary between patients and at different stages of the disease [21,22].

FRDA is one disorder with neurological and non-neurological involvement. Main non-neurological involvement includes the following: (i) cardiomyopathy, which is present in 85% of FRDA patients, and echocardiography and cardiac MRI are the most reliable techniques to quantify the progression of this condition [23]; (ii) kyphoscoliosis, which develops in most FRDA patients and ion many cases required surgical correction; (iii) pes cavus, which is also a frequent manifestation of this pathology [8]; (iv) cognitive impairment, which have been described in advanced stages of the disease [24] but is preserved during the early stages [25]; (v) diabetes mellitus, which occurs in approximately 25% of cases due to a combination of insulin deficiency and insulin resistance, with the involvement of pancreatic beta cells [26]; (vi) hypoacusis (hearing loss) and/or deafness; and (vii) visual system abnormalities, such as optic atrophy and eye movement anomalies, among others. The latter of which will be extensively discussed in a later section.

### 1.3. Genetics

FRDA is a neurodegenerative disease caused by mutations in the GAA triplet expansion in the first intron of the *FXN* gene on chromosome 9 (9q13–q1.1) [1,2,3,4,5,6,7,10]. The gold standard of genetic testing for FRDA is Southern blot. Short polymerase chain reaction (PCR) is used for the detection of alleles in the normal range, and long PCR is used for expanded alleles [27]. In addition, triplet repeat primed PCR (TP-PCR) [28] could be used, providing a characteristic peak pattern that confirms the existence of expanded triplet repeats in the FRDA gene [27].

Normal alleles have less than 33 consecutive GAA repeats in this gene, while abnormal alleles carry 66 to 1500 GAA repeats [2]. This expansion produces, as we have seen above, an altered iron uptake into the mitochondria, which results in an increased tissue vulnerability to oxidative stress [1,7,11,12,13,21,22]. Most people with FRDA are homozygous for this GAA trinucleotide expansion in intron 1 of the *frataxin* gene, and only 2–5% of them are compound heterozygous [7,9,11,12,13,21,22]. Age of onset and death in FRDA is related to the length of the GAA expansion, which is shorter in homozygotes [23,29]. There is a relationship between the length of the triplet repeat (GAA), *FXN* levels, and disease severity. Table 1 refers to the main neuro-ophthalmological clinical symptoms associated with the mean length of GAA repeat expansions. With short triplet repeats (although pathological), there is little *FXN* production, resulting in milder disease and later onset [30]. No patients with two abnormal *FXN* alleles and no clinical phenotype of FRDA have been found. No clinical symptoms have been found in carriers either. However, it is doubtful whether carriers are more susceptible to scoliosis and diabetes [31,32]. Among patients with two expanded *FXN* alleles, there may be modifiers that change *FXN* levels, either in a tissue-specific or global manner [25]. Thus, there may be risk factors or epigenetic processes that modify the course of the disease, such as DNA methylation and histone deacetylase activation [33,34]. Patients with point mutations may have a more frequent occurrence of atypical features, such as reflex retention and visual and hearing impairment [9,34,35,36]. The majority of point mutations, including those at RNA splice points, in the initiation codon and big deletions and nonsense mutations, do not produce functional proteins. Therefore it is, possible that these mutations are related to more serious phenotypes, such as diabetes and vision and hearing impairment, simply because they produce a less functional *FXN* [36,37]. Therefore, the amount of *FXN* and its function in different cells may be of great importance in defining the pathophysiological phenotype. However, other factors than the repeat length, as genetic, epigenetic and environmental variables, were suggested could play a role in determining the severity of disease [38].

## 2. Friedreich Ataxia and Eye

**Figure 1:**
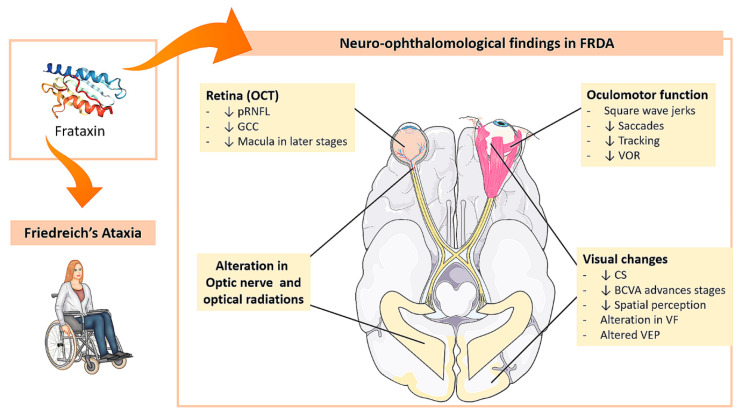
**Neuro-ophthalmological findings in FRDA**. FRDA: Friedreich’s ataxia, OCT: optical coherence tomography, pRNFL: peripapillary retinal nerve fiber layer, GCC: ganglion cell complex, VOR: vestibulo-ocular reflexes, CS: contrast sensitivity, BCVA: best-corrected visual acuity, VF: visual field, VEP: visual evoked potential. Images modified from https://smart.servier.com/ (accessed on 21 July 2021).

The majority of FRDA patients have no symptomatic visual impairment; however, up to 73% may have one or more clinical neuro-ophthalmological abnormalities [39,40], with optic atrophy being found in up to 30% of cases. The best-characterized ocular manifestations are extrinsic ocular motility disorders, but others can be found, such as optic neuropathy, optic radiation impairment, and, less frequently, retinitis pigmentosa-like syndrome [11,14,22,23]. As observed by diffusion-weighted imaging (DWI) performed in cerebral parenchyma, a large proportion of patients with FRDA show slow and progressive degeneration of the optic nerve and optic radiations, whereby both the anterior and posterior visual pathways are involved in this process [11]. In some cases, with a severe form of the disease and a large triplet expansion, the visual disturbance is subacute/acute, resembling Leber’s hereditary optic neuropathy [41,42]. All neuro-ophthalmological changes are summarized in Figure 1.

### 2.1. Oculomotor Function Alterations

There is a generalized dysfunction of the ocular motor system in FRDA. Extrinsic ocular motility disturbances are best characterized as they reflect disruption of the circuit from the brainstem to the cerebellum. Major findings include: (i) saccadic movement dysmetria, (ii) fixation instability with frequent square wave jerks, (iii) disruption of tracking movements, and (iv) vestibular abnormalities [11,13,43,44,45,46,47,48,49,50] (Table 2, Figure 1).

#### 2.1.1. Saccadic Movements

Saccadic intrusions and prolonged saccadic latency are the most typical abnormalities in FRDA patients [44], including hypo- and hypermetria, associated with slowed smooth tracking with superimposed fixation instability [48,50,59].

Among these movements, the most typical in FRDA are saccadic intrusions and prolonged saccadic latency [44]. Saccadic disturbances include hypometria and hypermetria and slowed smooth tracking with superimposed fixation instability [47,49,58].

- Saccadic latency

In FRDA patients, there is a prolongation of saccadic latency that correlates directly with latency variability [44]. In addition, a correlation has been found between the average latency and total score on the Friedreich Ataxia Rating Scale (FARS) [44,47]. This alteration could be explained because there are alterations in projections from frontal areas to the superior colliculus via the caudate and pars reticulata of the substantia nigra, which was described in another neurodegenerative disease [60], and also these changes could be replicated in FRDA. However, alterations in the brainstem or the interaction between burst and pause cells could also play a role [44].

- Saccade velocity

In FRDA patients, the average saccade velocity is affected as the disease progresses [44].

- Saccadic accuracy

Contrary to healthy people, who have a tendency for hypometry, in FRDA patients, there is a tendency for hypermetry [61]. Specifically, in FRDA, 54% of saccades were accurate within 10% of saccadic amplitude, 37% were hypermetric, and 9% were hypometric [44]. The same individual could have even hypermetria and hypometria [44]. The cerebellum and the dentate nucleus are affected in FRDA, as demonstrated by pathological and radiological studies [58], and the cerebellum is crucial for saccadic accuracy [59]. However, this disturbance may also originate from the pontine reticular formation, the superior colliculus, or from superior influences on these structures [44].

#### 2.1.2. Fixation

FRDA patients have fixation instability with frequent square wave jerks [46,48].

- Nystagmus

In 60% of FRDA patients, nystagmus caused by horizontal gaze occurs [44] [44], and also spontaneous vertical nystagmus in the dark was observed in 45% of the patients studied that correlated with the duration of the disease [44].

- Square wave saccades

Square wave saccades are inappropriate saccades that take the eye off the target, followed by a nearly normal intersaccadic interval (approximately 200 msec), and then a corrective saccade that brings the eye back to the target [62]. Square wave saccades are characteristic of FRDA [44], and a possible explanation could be due to a loss of inhibition in the paramedian pontine reticular formation (PPRF) [63,64]. In FRDA, alterations in saccades and fixation are related to dysfunction of the superior colliculus-omnipausal neuron pathway [44] that produces bilateral tonic inhibition to the PPRF via the omnipausal neurons, which exerts a tonic inhibitory effect on the saccadic neurons in order to keep the eye on target [65]. However, the cerebellum and the frontal and parietal areas of the visual pathway, which have a straight route to the superior colliculus, could also be involved in the eye motility disturbances of this disease [62,66].

With a target-on (a fixation point), FRDA patients have lower square wave saccades amplitude and duration than a target-off [44]. Moreover, the frequency of these saccades correlated inversely with the age of onset but not with the duration of the disease. Patients with prior disease outbreaks had more frequent and shorter square wave saccades and greater fixation instability. The number of square wave saccades can also be considered as an indicator of FRDA severity, given the high correlation between the FARS score and the appearance of these macro-saccades [44].

In addition, variations in the morphology and duration of square wave jerks are found, which may also sometimes be mixed with eye flutter. Eye flutter is characterized by rapid, dysmetric horizontal oscillations that may occur in the primary eye position due to loss of control of eye posture maintenance by the cerebellum [44]. In a patient with progressive ataxia, the presence of ocular flutter may indicate FRDA [67], being this alteration present in later stages of FRDA [66]. Both square wave saccades and eye flutter can also occur in other processes such as demyelination, infection, drugs, etc. [62]. The oscillations may be produced by the brainstem circuits and not by the cerebellum [68]. However, it is possible that cerebellar pathology may cause an imbalance in the saccadic system because of its relationship to the brainstem [44]. If this occurs, square wave saccades and ocular flutter could represent different expressions of the same pathologic process [44].

Although square wave saccades are classically characterized as appearing in the horizontal plane [66] and are the most common in FRDA, some patients may exhibit oblique square wave saccades with a prominent vertical component, and occasionally, some patients exhibit purely vertical saccades [44]. However, no vertical eye flutter is observed. Vertical components have been identified in almost half of FRDA patients, although their existence does not correlate with other eye movement anomalies [44]. In patients with FRDA, square wave saccades with a vertical component have been associated with lesions in the superior colliculus [44,65,69].

#### 2.1.3. Tracking Movements

In all individuals with FRDA, tracking movements are abnormal, with recurrent saccadic intermissions, small pursuit achievement, and recovery saccades [44].

#### 2.1.4. Vestibulo-Ocular Reflexes (VOR)

In patients with FRDA, oculography measurements have found alterations of the VOR and visual-vestibular interactions [48,70]. In addition, alterations in caloric tests have also been noticed in most patients with this pathology [46,67,71]. In FRDA, the latency and gain of the VORs are altered, producing a consistent and severe bilateral vestibulopathy with disruption of all six semicircular canals. These data are compatible with injury to the vestibular end organ, its nerve, or its brainstem nucleus [44]. In contrast to certain forms of cerebellar ataxia, particularly when accompanied by cerebellar atrophy, FRDA patients are noticed to have markedly reduced VOR gain [44]. Thus in FRDA, the considerable vestibulopathy serves to differentiate it from the other cerebellar ataxias in which normal or heightening VOR gain is found [67,72]. Oscillopsia, which is caused by loss of the VOR bilaterally [73], is not a predominant symptom in FRDA [44].

#### 2.1.5. Clinical Utility of Ocular Motility Testing in Patients with FRDA

FRDA patients have a severe two-sided VOR impairment and normal saccadic velocity, which is a clinical hallmark of FRDA and distinguishes it from several spinocerebellar ataxias of dominant inheritance; thus, these eye movements (saccadic latency and VOR) could be used as biomarkers for this disease [44,47].

In summary, FRDA causes severe and extensive disorders of the oculomotor system, leading to alterations in vision and quality of life. Alterations in eye motility can be proved precisely and faithfully, displaying gradual alteration that correlates with other markers of illness hardness, and consequently, these alterations are encouraging as measures for following up the FRDA progression [74,75].

### 2.2. Visual Pathway Disorders

#### 2.2.1. Visual Evoked Potentials (VEP)

About 60–90% of patients with FRDA have binocular disturbances on VEPs [11,39,43,51]. Patients with the most serious grades of visual deterioration (optic atrophy) have plain or undetectable VEPs, while in less serious patients, the outcomes are delayed, reduced in amplitude, and show a greater degree of temporal dispersion, which has pathophysiological significance and increases the need for FRDA heterogeneity interrogation [40,52]. In general, P100 amplitude is reduced, especially in patients who have latencies below 115 ms (upper limit of normal), as long as there is a significant inverse correlation between P100 amplitude and latency in those patients with latencies over the normal range. Most patients with identifiable responses, even those with prolonged latencies, are normal with respect to waveform, time dispersion, and interocular differences [39]. VEP patterns correlate with the age of disease onset and International Cooperative Ataxia Rating Scale (ICARS) scores [11]. A suitable correlation between VEPs and clinical neuro-ophthalmological findings has been described. Temporal optic disk pallor is most frequently associated with abnormal VEPs. However, if no abnormalities in VEP are found, no abnormalities in color vision or visual acuity are usually found. [39]. Pattern-reversal VEPs are useful in differentiating genetic ataxias and spinal degenerations and may be a useful variable in distinguishing these pathologies from multiple sclerosis [51].

Most FRDA patients are asymptomatic, although optic nerve lesions are not uncommon, and when this occurs, they are noticeable by the clinical and the VEP. Furthermore, in FRDA patients, the VEP alteration is reliable with gradual nerve fiber loss and associated conduction delaying, showing that the visual pathway is impaired by the same extensive process of axonal degeneration found far and wide in the nervous system [39,52] (Table 2).

#### 2.2.2. Electroretinogram (ERG)

In general, ERGs in patients with FRDA are normal or with minimal alterations, suggesting more secondary than primary retinal involvement [39]. Moreover, in pattern electroretinogram (PERG), a moderate amplitude reduction with normal latencies has been described and associated with moderated and diffuse fiber reduction by red-free light retinography [43] (Table 2).

#### 2.2.3. Contrast Sensitivity (CS)

Patients with FRDA have worse scores on the low-contrast Sloan Letter Table, remarkably in 1.25% and 0.6% charts, contrasted with controls [53]. However, the ability to distinguish between FRDA patients and controls using this test is higher for the 5% and 1.25% charts [53]. In a longitudinal study with a mean follow-up of 4.4 years, visual acuity measured with low-contrast Sloan Letter Table at 2 m (Precision Vision, LaSalle, IL, USA) decreased significantly at 2.5% contrast (−0.81 letters/year) and 1.25% contrast (−1.12 letters/year). There is a relation between time and GAA triplet repeat length; the rate of visual acuity loss at 2.5% and 1.25% contrast is worse in patients with higher GAA triplet repeat lengths. Consequently, low-contrast visual acuity drop-off linearly with time in FRDA, especially at longer GAA repeat lengths [56]. Low-contrast Sloan charts may have a possible role in evaluating the degree of the illness and visual function in FRDA patients [53], analogous to their proposed role in multiple sclerosis [54]. Therefore, contrast sensitivity evaluation could serve as a potential biomarker in patients with FRDA [56] (Table 2).

#### 2.2.4. Spatial Perception

In one study, a battery of neuropsychological tests was performed on a cohort of FRDA patients with normal visual acuity. The patients showed impairments in tests of spatial construction and conceptual perception. According to the authors, these alterations could be because cerebellar structures may be involved in certain spatial tasks, and the lack of action or training in this space as a consequence of the disease could explain the deficits [57] (Table 2, Figure 1).

#### 2.2.5. Best-Corrected Visual Acuity (BCVA)

In FRDA, the axons of the papillary-macular bundle, which constitute the anatomical substrate of central vision, color, and high spatial frequency contrast sensitivity [12,76,77,78], seem to be preserved [11,12,13,55]. Loss of visual acuity is therefore uncommon [11,53] or occurs at later stages of the disease [4,11,22,55,79]. Over an average of 4.4 years of follow-up, visual acuity at 100% contrast decreases (−0.37 letters/year) [56]. Reduced BCVA can range from moderate to severe and is produced by optic atrophy, retinal degeneration, or both [50] (Table 2, Figure 1).

#### 2.2.6. Visual Field (VF)

Three patterns of affectation have been described in the VF of patients with FRDA, ranging from severe and concentric reduction in sensitivity in later stages, pursued by concentric upper and/or lower defects in mild ones, until reduced sensitivity in a paracentral area in the earliest stages [4,11,79]. However, central vision is usually spared in most FRDA patients in the early stages of the disease but deteriorates with disease progression [79]. One study also analyzed the correlation between VF and neurological disability (Scale for the Assessment and Rating of Ataxia: SARA), finding that the index of visual field and mean deviation had a slight negative correlation with the SARA score [79] (Table 2, Figure 1).

#### 2.2.7. Optical Coherence Tomography (OCT)

There are very few studies using OCT in patients with FRDA [4,11,22,55,80] and only one that included OCT follow-up [79] (Table 3, Figure 1).

- Peripapillary retinal nerve fiber layer (pRNFL)

All OCT studies showed a statistically significant decrease in the mean thickness of the peripapillary retinal nerve fiber layer (pRNFL) compared to the control group at baseline scanning [4,11,22,55,79]. The groups of Seyer et al. [22] and previous work by our group [79] also analyzed the quadrants of the pRNFL, finding that, in patients with FRDA, a statistically significant decrease in thickness was observed in all quadrants of the pRNFL with respect to controls, which from the highest to the lowest degree of involvement corresponded to the inferior, superior, nasal, and temporal papillomacular bundle. Only our group [79] analyzed the pRNFL by hourly sectors, finding that all the hourly sectors of the pRNFL were statistically decreased with respect to the control group, both in the baseline and follow-up examinations, except for the H8 sector in the baseline examination. On the other hand, a comparison between baseline and follow-up examinations in patients with FRDA showed a significant decrease in the H7 sector during follow-up, which could correspond to disease progression. The study of the pRNFL by sector could be important due to the retinotopic distribution of retinal axons [81,82,83].

OCT findings have also been correlated with neurological disability measured on different clinical scales. Thus, Fortuna et al. [11], Noval et al. [55], and Dağ et al. [4] correlated the mean thickness of the pRNFL with the ICARS scale, Noval et al. [55] and Seyer et al. [22] correlated the mean pRNFL thickness with FARS, and Thomas-Black et al. [80] and previous work by our group [79] correlated mean pRNFL thickness with SARA. The average pRNFL thickness and the age of onset of FRDA were found to be correlated [11,22,80].

It was found that there are multiple correlations between OCT parameters and SARA, especially in the temporal and inferior quadrants and sectors H7–H11 of the pRNFL [79]. These authors found less involvement in the temporal quadrant and the H8 sector, corresponding to the papillomacular bundle (which corresponds to the parvocellular system), and thought that this might explain why the BCVA is not affected until later stages of the disease [79]. Since there is mainly involvement of the superior and inferior quadrants of the pRNFL, it would suggest a preferential contribution from retinal parasol ganglion cells, which project to the magnocellular pathway (M cells). Therefore, these neurons are located outside the macula and would not contribute specifically to BCVA [84].

Furthermore, previous work by our group [79] found in the aROC curve analysis that the best parameter for distinguishing between FRDA patients and controls was the mean pRNFL thickness, with a cut-off point of 80.5 µm (sensitivity = 100%; specificity = 87.5%; AUC = 0.984). Values below this would correspond to patients with FRDA. Thus, the pRNFL average thickness would be a parameter with excellent diagnostic capability.

- Ganglion Cell Complex (GCC)

Only two studies analyzed the GCC. Dağ et al. [4] found a decrease in the GCC average thickness in the superior and inferior macular areas in FRDA patients. Meanwhile, a study published by our group [79] divided the superior and inferior macular regions into six areas, and all of them, except for the supero-temporal area, showed a reduced thickness in FRDA patients compared to control at baseline examination. Six months later, they showed a decrease in all GCC thickness areas in the FRDA cohort than the control group. Moreover, when they compared FRDA follow-up patients compared to their baseline examination, most GCC areas also showed a significant decrease. They thought that it could correspond to disease progression [79]. This GCC decrease in the OCT could be produced by a general involvement of retinal ganglion cells, specific targets of mitochondrial-mediated neurodegeneration [11,42,76,77]. In FRDA, the axons of the ganglion cells would be affected first because they have more mitochondria, so the first changes described are in the pRNFL.

Only our group [79] studied the correlation between the GCC and the SARA, finding that most of its measurements had a moderate negative correlation with the SARA scale; therefore, patients with a greater neurological disability had lower GCC thickness.

- Macula

Just a few OCT studies have analyzed what occurs in the macula in FRDA. Noval et al. [55] found that foveal thickness and macular volume were normal. In contrast, Dağ et al. [4] described a foveal thickness decrease, and Seyer et al. [22] observed a decrease in macular thickness measurement. Both authors correlated this thinning with the progression of the disease. In previous work, a normal foveal thickness was documented on baseline and follow-up examinations [79]. However, in both explorations, several areas were thinned compared to the control. Most notably, the follow-up study found that the decrease in macular thicknesses progressed significantly in different macular areas, so it appears that the FRDA affects the macula [79]. The differences found between the various studies could be related to the duration of the disease. Table 3 shows in detail the different OCT studies performed in FRDA patients. It described the population with different age ranges: younger populations describe only peripapillary involvement, while in older populations, macular thickness is also found to be affected.

These results advocate that those macular changes measured by OCT could be a characteristic of later stages of FRDA and could be implicated in the visual impairment in these patients. Macular changes could therefore help to assess the progression of FRDA [22].

Our group [79] described a correlation between macular thickness and neurological disability measured by SARA score by dividing the macula into different areas, including foveal thickness, by analyzing the macular volume. Describing an inverse, significant and mild correlation in the superior and temporal areas of the inner macular ring in both baseline and follow-up examinations, and in the superior inner macular ring (IMR), cubic volume, and mean cubic thickness in the follow-up examination. Therefore, the authors concluded that a lower macular thickness corresponds to a higher neurological disability as measured by the SARA score.

**Figure 2:**
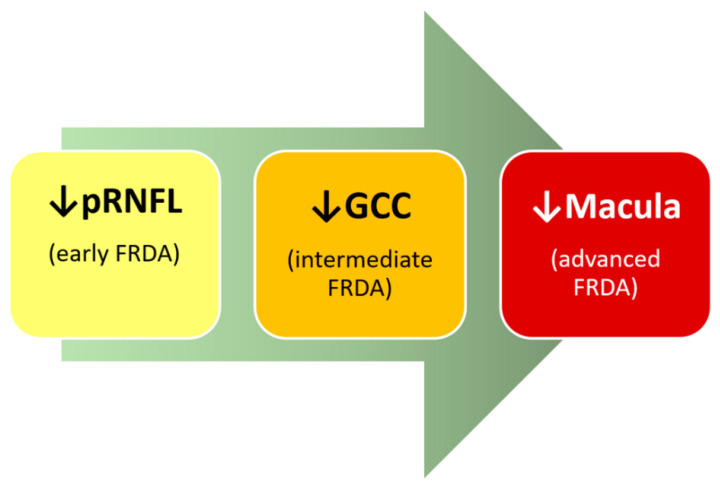
**OCT and FRDA: summarize the sequential involvement.** In the early onset of FRDA, there is an affectation of pRNFL; in the intermediate FRDA, there is a loss in the ganglion cell complex (GCC); and in advanced stages, there is a thinning in the macular area.

In FRDA, there would be a sequential involvement that would be measurable by OCT. Firstly, the decrease in the thickness of the pRNFL would be the main event observed in these patients. Secondly, there would be a loss of the ganglion cell soma induced by axonal death, and thirdly, all this could lead to the macular thinning that is observed late in the course of the disease. Therefore, FRDA could alter both axons and ganglion cell somas, probably due to retinal axonopathy and neuronopathy (Figure 2) [22].

It could be concluded that the parameters of the OCT could be considered an objective biomarker in FRDA. As we have seen, there are several parameters, but above all, the mean pRNFL thickness correlates with a measure of clinical severity and the clinical scales of neurological disability. In addition, OCT enables a direct measurement of the affected neural tissue, allows disease progression follow-up, and could be suitable for FRDA multicenter trials.

#### 2.2.8. Histopathological Studies of the Retina of Patients and Experimental Models of FRDA

Work that analyzes retinal histological changes in FRDA is scarce, especially that using patient tissues. As we have seen previously, in FRDA, retinal ganglion cells (RGCs), retinal pigment epithelium (RPE) cells, and photoreceptors can be altered. Moreover, these alterations can produce ophthalmological manifestations such as retinitis pigmentosa-like and optic neuropathy. In the study by Crombie et al. [85], optic atrophy and loss of RPE cells were found in human tissues from FRDA patients; however, they did not find morphological changes or pathology of RPE. Furthermore, the analysis of induced pluripotent stem cells (iPSCs) derived from RPE cells from FRDA patients showed no significant oxidative phosphorylation activity or phagocytosis changes. Retinas of animal mouse models with FRDA were also analyzed in this study [85]. These models, YG22R and YG8R, contained human *FXN* but did not recapitulate observations found in the human retina, such as ganglion cell loss.

Another study was performed in a mouse model of FRDA based on the knock-down of frataxin by RNA interference to make FRDAkd mice [86]. In this model, the mice developed abnormal mitochondria and had cardiac and nervous system alterations that paralleled those seen in FRDA patients. In this study, the retina of FRDAkd animals was analyzed by transmission electron microscopy, and photoreceptor disruption and a significant increase in degenerated RPE cells were found. As photoreceptor alteration is correlated with visual impairment [87], the results of Chandran et al. may suggest that *FXN* deletion may be related to retinal neuronal degeneration [86].

## 3. Material and Methods

**Figure 3:**
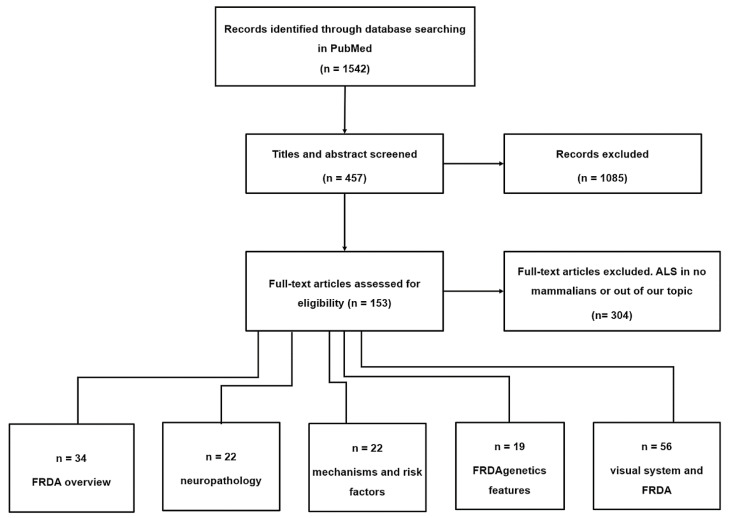
Flow chart materials and methods. Abbreviations: ALS: Amyotrophic lateral sclerosis; FRDA: Friedreich’s ataxia.

In this study, literature searches up to April 2021 was carried out using the term “MESH” in PubMed, and the following keywords and word combinations: “Friedreich ataxia” AND “mitochondria”, “oxidative stress”, “Iron”, “Microglia”, “Genetics”, “Oculomotor”, “Visual Pathways”, “Evoked Potentials”, “Visual”, “Contrast Sensitivity”, “Visual Fields”, “Visual Field Tests”, “Visual Acuity”, “Retina”, OR “Optical Coherence Tomography”. After the initial search, the articles were filtered by the author’s criteria, English or Spanish language, and a relationship between FRDA and the visual pathway as a priority topic. Thus, 81 articles met the requirements, while 304 were excluded (Figure 3). In more detail, the following inclusion criteria were used in the selection of articles: (i) articles describing the general pathology of FRDA; (ii) papers establishing a relationship between alterations in the visual system and FRDA; (iii) papers on retinal alterations in both animal models and patients with FRDA; and (iv) articles containing human clinical trial studies with optical coherence tomography (OCT) analysis. The criteria used for excluding articles were: (i) papers that had not been performed in animal models of mammalian FRDA and (ii) articles whose subject matter was not closely related to the objective of this review or that did not satisfy the selection requirements indicated by the authors.

## 4. Conclusions

In FRDA, there is visual impairment, both in motor function and in the anterior and posterior visual pathways. The earliest detectable alterations occur at the oculomotor level and in contrast sensitivity tests. However, as the disease progresses, both neurophysiological tests and functional visual tests such as VA and VF show alterations. New techniques such as OCT can detect changes in the visual pathway and its progression over time. Retinal structural changes in FRDA would indicate that peripheral neurological diseases may also have retinal involvement. These changes may have great sensitivity as a biomarker of susceptibility/risk in this pathology, even though retinal alterations do not produce clinical visual symptoms until the late stages of the disease. In conclusion, in FRDA patients, the use of OCT could be recommended for further investigations into diagnostic and follow-up techniques for this disease.

## Figures and Tables

**Table 1:** Neuro-ophthalmological clinical symptoms associated with a mean length of GAA repeat expansions.

Clinical Symptoms	Mean Length of GAA Repeat Expansions	References
Horizontal nystagmus	Between 630 ± 230 and 890 ± 230	[29]
Saccadic-pursuit eye movements		
Reduced visual acuity		
Axonal neuropathy		
Abnormal visual evoked potentials		

Abbreviations: GAA: guanine-adenine-adenine.

**Table 2:** Neuro-ophthalmological findings in FRDA.

Changes in FRDA Patients	Main Features	References
Oculomotor function	Dysmetria in saccadic movements. Interruption of tracking movements. Vestibular ocular reflexes altered. Fixation instability with frequent square wave jerks	[11,13,43,45,46,47,48,49,50]
Neurophysiology	VEP: altered	[11,39,43,51,52]
	ERG: Usually normal or minimally abnormal	[39,43]
Color vision	Normal	[39]
Contrast sensitivity	Supplementary role of low-contrast Sloan Chart testing in the assessment of disease status and visual function in FRDA	[53,54]
	Correlation with mean pRNFL thickness and binocular VA with 1.25% and 2.5% contrast	[55]
	Low-contrast visual acuity drop-off linearly with time in FRDA, especially at longer GAA repeat lengths	[56]
Spatial perception	Impairment in spatial construction tests	[57]
Visual acuity	Loss of visual acuity is rare	[11,53]
	30% of cases with optic nerve atrophy	[16]
	Chronic and progressive impairment, late-stage effects occur	[4,11,14,23,58]
	Correlation with mean pRNFL thickness and high-contrast VA	[55]
	Reduced BCVA can range from mild to severe and is caused by optic atrophy, retinal degeneration, or both	[50]
	There is a subgroup mimicking Leber’s hereditary optic neuropathy with severe BCVA affectation	[41,42]
Visual field	Three patterns of involvement ranging from reduced sensitivity in a paracentral area, followed by concentric superior and/or inferior defects, to a general and concentric reduction in sensitivity in later stages	[4,11,58]
OCT	In detail in Table 3	
Visual pathway involvement	Retina: retinosis pigmentaria-like syndrome	[11,14,22,23]
	Anterior (optic nerve)	[11]
	Posterior (optical radiations)	

Abbreviations: VEP: Visual evoked potentials; ERG: electroretinogram; pRNFL: peripapillary retinal nerve fiber layer; VA: visual acuity; GAA: guanine-adenine-adenine; BCVA: best-corrected visual acuity; OCT: optical coherence tomography.

**Table 3:** Studies with OCT in FRDA patients.

Author	Study Type	OCT	Mean Age ± SD	pRNFL	Macular Thickness	GCC	Correlation OCT with Neurological Disability (Clinical Scale)
Fortuna et al. 2009 [11]	Cross-sectional study	TD- Stratus	32.00 ± 8.00	↓ mean RNFL	Not documented	Not documented	pRNFL with ICARS r = –0.576
				↓ 4 quadrants			
Noval et al. 2012 [55]	Cross-sectional study	TD- Stratus	25.22 ± 6.69	↓ 75% pRNFL	Normal foveal thickness and macular volume	Not documented	pRNFL with ICARS (RE r = 0.638 and LE r = 0.695)
				Normal temporal quadrant			pRNFL with FARS (RE r = 0.531 and LE non-significant)
Seyer et al. 2013 [22]	Cross-sectional study	TD- Stratus for RNFL	28.20 ± 15.90	↓ pRNFL	Not documented	Not documented	pRNFL with FARS (r = −0.72)
				↓ 4 quadrants			
		SD-Cirrus for macula		Not documented	↓ Macular thickness (20.7%)	Not documented	Not documented
Dağ et al. 2014 [4]	Cross-sectional study	SD RS-3000	32.10 ± 10.46	↓ pRNFL	↓ CMT	↓GCC (S and I)	pRNFL with ICARS (r not documented)
				↓ 4 quadrants			
Thomas-Black et al. 2019 [80]	Cross-sectional study	TD-Stratus	32.0 ± 11.80	Values not compared with control	Not documented	Not documented	pRNFL with SARA r = −0.457
Rojas et al. 2020 [79]	6-month follow-up study	SD-Cirrus	35.00 ± 10.36	↓ RNFL	Normal CMT	↓GCC	Several OCT parameters with SARA (most relevants):
				↓ 4 quadrants (I > S > N > T)	↓ Macular thickness (IMR: S, N, I; OMR: N, Cube Vol.)		pRNFL (r = −0.693), T-Q (r = −0.803); H10 (r = −0.783)
				↓ 4 H5-H11			AMI-S (r = −0.507)
							GCC [I-T (r = −0.679)]

Abbreviations: ↓: decrease; OCT: optical coherence tomography; TD-OCT: temporal domain OCT; SD-OCT: spectral-domain OCT; SD standard deviation; pRNFL: peripapillary retinal nerve fiber layer; CMT: central macular thickness; IMR: inner macular ring; OMR: outer macular ring; GCC: ganglion cell complex; ICARS: International Cooperative Ataxia Rating Scale; FARS: Friedreich Ataxia Rating Scale; SARA: Scale for the Assessment and Rating of Ataxia.

## Data Availability

All data reported in this paper are available in the cited references, available either at pubmed.gov or at the websites within the citations.
